# Multivariate Analysis of the Predicted Probability of Smoking Behavior of Foster Care Minors: Results of a Study from Romania

**DOI:** 10.3390/ijerph19031173

**Published:** 2022-01-21

**Authors:** Corina Eugenia Budin, Anca Diana Maierean, Ioana Roxana Bordea, Iuliu Gabriel Cocuz, Liviu Sorin Enache, Elena Luminita Enache, Damiana Maria Vulturar, Ana Chis, Doina Adina Todea

**Affiliations:** 1 Department of Pathophysiology, University of Medicine, Pharmacy, Science and Technology Târgu Mureș, 540139 Targu Mures, Romania; cora_bud@yahoo.com (C.E.B.); iuliuco@gmail.com (I.G.C.); 2 Clinical County Hospital Mureș, 540142 Targu Mures, Romania; 3 Department of Pneumology, University of Medicine and Pharmacy “Iuliu Hațieganu” Cluj Napoca, 400012 Cluj Napoca, Romania; anca.lupascu91@gmail.com (A.D.M.); damiana_vulturar@yahoo.com (D.M.V.); anna_f_rebrean@yahoo.com (A.C.); doina_adina@yahoo.com (D.A.T.); 4 Department of Oral Rehabilitation, University of Medicine and Pharmacy “Iuliu Hațieganu” Cluj Napoca, 400012 Cluj Napoca, Romania; 5 Emergency Clinical Hospital Târgu Mureș, 540136 Targu Mures, Romania; enachesliviu@yahoo.com; 6 Dimitrie Cantemir University, 540545 Targu Mures, Romania; dincaluminita@yahoo.com

**Keywords:** tobacco use, smoking behavior, foster care minors

## Abstract

Background: There are a multitude of factors that influence smoking status, and minors from the social protection system are a vulnerable category in terms of smoking. Methods: The objective of this research was to assess the degree of smoking dependence and to identify potential predictors of smoking status in foster care teenagers. Smoker status was confirmed by dosing CO in the exhaled air, and the degree of dependence was assessed using the Fagerström score. We performed a multivariate logistic regression analysis. Results: From the 275 foster care minors, 22.5% were current smokers. Exhaled CO was not influenced by general demographic factors, was associated with the frequency of smoking, and was positively correlated with the estimated number of cigarettes consumed daily and with the Fagerström score. The calculated probability of being a smoker was less than 20.4% in 75% of nonsmokers, whereas 75% of actual smokers had a predicted probability higher than 30.3%. Conclusions: In addition to age, gender, social environment, previous exposure to secondhand smoking, and residential type of foster care system, the expressed opinions regarding the health effects of tobacco use were associated with smoking in foster care teenagers.

## 1. Introduction

Tobacco dependence is a chronic disease that usually starts during adolescence and is considered to be a public-health priority [1]. All health professionals should consider tobacco consumption (both by inhalation and chewing) as an important health hazard, even if the patient has not yet developed tobacco dependence. As tobacco dependence is a disease, it should be diagnosed and treated as with any other chronic disease [2].

Banning the sale of tobacco products to minors, price raising, printed warnings on cigarette packs, and providing nonsmoking areas are crucial in preventing adolescent smoking [3]. Globally, at least 1 in 10 teenagers aged between 13 and 15 smoke, although there are areas where the starting age is even lower [4]. In developed countries with sustained antismoking programs, the frequency of smoking among minors appears to be declining [5].

Where smoking cessation measures fail, it is necessary to identify possible factors that turn a teenager trying their first cigarette into a daily smoker [6]. There are currently no gender differences in age at onset of smoking or in the number of active smokers among adolescents [7]. However, a signifiant percentage (40%) progress from the moment they smoked their first cigarette to the status of a daily smoker. This development can be determined by familial and extrafamilial factors. Thus, of the extra-familial factors, for boys, the most important influence was peer smoking; for girls, it was sibling smoking and media messages [6,7].

There are a multitude of factors that influence smoking status: age at onset, entourage, stress level, exposure to images from media, urban or rural environment, and school and family habits [6,8]. The risk factors for initiating smoking vary depending on the pattern of each country. The importance of this topic is highly significant in low and middle-income countries, because educational programs in these regions have been implemented recently [8].

Minors in the social protection system represent a vulnerable category in terms of smoking and smoking onset. The negative events that they experience in their life significantly increase the risk of becoming smokers [9,10]. In this social group, an increased prevalence of drug use was demonstrated over time, and smoking and alcohol were chronologically used first [11,12]. Given that these adolescents sometimes live in a more permissive or disinterested environment regarding smoking, the educational programs addressed to this social category are all the more important, in order to prevent the long-term effects on the health of these adolescents [10]. 

The hypotheses of our study were to assess the degree of nicotine dependence and to identify potential risk factors for smoking in a large number of foster care teenagers from two different counties in Romania.

Purpose of the study

Our study’s primary purpose was to identify potential predictors of smoking status in foster care teenagers from two counties in Romania. To reach this objective, we approached as a secondary outcome the degree of smoking dependence in this population. 

The practical importance of these applied measures will be found in the personalized and customized guidance of the educational programs on the topic of smoking addressed to adolescents within the social protection system in Romania.

## 2. Materials and Methods

This cross-sectional pilot study was conducted from October 2017 to December 2018 at the General Directorate for Child Protection in Mureș and Cluj counties, Romania. The study was approved by the Romanian Society of Pneumology in accordance with European and national legislation, and received approval number 443 from November 2016 from the Ethics Committee of the Iuliu Hațieganu University of Medicine and Pharmacy Cluj-Napoca, Romania. The entire methodology complied with the personal data protection protocol. Each participant agreed to be included in the study. The consent was signed both by the legal guardian and by the Director of the General Directorate for Child Protection (GDPC) in Mureș and Cluj. 

The General Directorate of Child Protection is the official institution at the administrative level in Romania with legal responsibility for minors registered in the social protection system. At the level of this institution, there is a database where minors are officially registered, both those included in the professional maternal assistance system and those included in the residential system or day-care system. The population included in the study was the total number of minors aged between 10 and 18 years of age coming from the maternity professional and residential care system, registered in this database of the General Directorate of Child Protection Mures and Cluj on 1 November 2016, who met the inclusion and exclusion criteria.

The study was carried out on the general population, represented by all institutionalized minors from Mures and Cluj counties aged between 10 and 18 years. This population was investigated through a qualitative and quantitative approach to assess the level of knowledge and health-harmful habits regarding tobacco use in adolescents. 

The study was conducted at the same location for all participants, a location belonging to the General Directorate of Child Protection, but to facilitate communication with them and travel to this location, they were divided into groups of 20–25 participants. Every meeting started with a communication from us on general concepts in tabacology, because these minors had no prior knowledge of this topic and had not participated in educational programs on the subject. They were subsequently given the questionnaires to complete. The concepts presented did not influence the answers to the questions in the questionnaire.

The qualitative approach was represented as a major objective of determining the actual frequency of smoking among this population, an interpretation that is the subject of another study [7]. The qualitative approach essentially assesses the perception of smoking and its negative effects on health at both an individual and a social level. Demographics, social factors, the views of minors, knowledge of tabacology or lack of it guided us in the selection of predictive factors (positive and negative) that influence smoking status.

Participants

Inclusion criteria. Our study included all the institutionalized minors from the Mureș and Cluj counties who were aged between 10 and 18, both smokers and nonsmokers, who were able to read and write in order to complete the questionnaire, and who had expressed their consent to participate in writing.

Exclusion criteria. From the study were excluded institutionalized minors who did not express their agreement to participate and institutionalized minors whose legal guardians did not provide written consent for participation.

All the participants, both those in the professional maternal assistance (PMA) and residential systems, were divided into equal and relatively heterogeneous groups of approximately 20 participants on the basis of territorial affiliation in order to facilitate their presentation at study locations, where they were accompanied by their legal guardian.

Prior to completing the questionnaires, the participants were informed as to the voluntary nature of their participation. Legal guardians were not present during the exhaled carbon monoxide (CO) measurement. The questionnaires were collected by the team of investigators, without the involvement of GDPC representatives.

The questionnaire was self-reported and included 38 questions (13 single-answer, 15 multi-answer, and 3 free-answer questions) on demographic characteristics, use of tobacco products (number of cigarettes per day, degree of nicotine dependence, knowledge of legislation related to smoking, exposure to secondhand smoke), adolescents’ perceptions of smoking, and 7 single-answer questions from the Fagerström test (only smokers answered) [2,7,13]. In total, 23 questions were about cigarette smoking and 8 questions were about e-cigarette use.

In this study, a smoker was considered to be a person who had smoked more than 100 cigarettes (including rolled cigarettes, cigars) in their lifetime and had smoked during the last 28 days prior to examination; an ex-smoker was defined as a person who had smoked more than 100 cigarettes in their lifetime, but who had not smoked during the last 28 days prior to the study; a nonsmoker was defined as a person who had not smoked more than 100 cigarettes and did not smoke at the time of the study [2].

Degree of nicotine dependence. The degree of nicotine dependence was determined using the internationally accepted Fagerström Test for Nicotine Dependence (FTND) questionnaire, adapted for adolescents [13,14]. The Fagerström test is a standard tool for determining the degree of physical dependence on nicotine. It contains seven questions that assess the number of consumed cigarettes, the urge to use tobacco products, and dependence. In the adolescent-adapted version, the questions are scored from 0 to 2. The final score can vary between 0 and 9. The higher the score, the stronger the addiction [13,14].

Quantification of exhaled CO. Exhaled CO was assessed with a Micro Smokerlyzer (Bedfont Scientific Ltd. England) device, commonly used in smoking-cessation programs or in smoking studies. The device features a module for teenagers. The display was adapted and changed for each profile, as needed. Disposable mouthpieces were used for each participant, in compliance with standard hygiene criteria [15]. Carbon monoxide was measured and expressed as parts per million (ppm). Although the cut-off value of 9 ppm is currently used for adults, we used the following reference values: nonsmoker (0–4 ppm), danger/transition zone (5–6 ppm), smoker (7–10 ppm), frequent smoker (11–15 ppm), heavy smoker (16–25 ppm), and high-risk smoker (26–35 ppm, >36 ppm), as set in the adolescent profile module of the measuring device (Micro Smokerlyzer, Operating Manual) [15].

In order to not change their smoking behavior and lead to the erroneous recording of parameters, the participants were not informed about the measurements to be performed until the morning of the study.

**Statistical analysis**. The data were centralized in Microsoft Excel spreadsheets. A statistical analysis was performed in the R statistical environment (version 4.0.3, cran.r-project.org, accessed on 10 December 2020). The statistical significance threshold was set to 0.05.

In order to identify factors associated with current smoker status in institutionalized children, we performed a multivariate logistic regression analysis. Records wherein smoking status was not specified were excluded from the analysis.

The potential predictors were chosen on the basis of the knowledge of the study subject and following selection through the univariate analysis of their association with smoking status. In the univariate analysis, we chose a permissive selection threshold, with *p* < 0.5, according to the number of participants. Therefore, the multivariate analysis included demographic factors (age, sex, place of origin), environmental factors (existence of smoking parents in the family, exposure to secondhand smoke), and aspects related to the personal beliefs of the participants (expressed by answers to questions related to the harmful nature of smoking and secondhand smoke, awareness of the health risks associated with smoking, possible effects of smoking cessation, and the perceived effects of e-cigarettes).

In our understanding, asking a teenager what he believes about a behavior that may affect him (in this case, smoking), and his motivation, is a form of human input. It is also one of the qualitative approaches used in this study.

For the multivariate analysis, the missing values of the selected variables were filled by multiple imputation (chained-equations method), with logistic regression for binary variables, multinomial logistic regression for unordered categorical variables, proportional odds model for ordered categorical variables, and predictive mean matching for continuous variables [16]. In total, we performed 300 imputation cycles, with 5 repetitions for each cycle. For each cycle of 5 imputations, the average of the imputed values was calculated for continuous variables, whereas the majority value was chosen for categorical variables. Next, on the basis of the logistic regression model, the coefficient for each predictor of smoking status was calculated. The procedure was repeated for each cycle and the predictive model included the coefficients of each predictor averaged over the 300 cycles.

Predictive variables for smoker status were selected by successive exclusion, starting with a predictive model that included all the potential predictors initially chosen by univariate analysis, gradually excluding one predictor at a time (the predictor with the lowest probability of significance, i.e., the highest *p* value) and checking whether the model without the predictor in question demonstrated significantly lower predictive power than that of the model that included that predictor. The selection of predictors was completed when the exclusion of any variable from the predictive model led to a statistically significant decrease in the predictive power of the model for smoker status.

We understand that variable selection based on *p*-value, as is the case with stepwise selection with backward elimination, implies risks (like any selection process)—one of which is the elimination of a variable that might be associated with the outcome but, due to chance, did not reach a certain “significance” threshold. 

However, variable selection is needed to some degree, in order to limit the risk of the model overfitting the data (i.e., the risk that the model comprising a large number of variables describes the studied individuals so well that it is not translatable to other groups from the same population). We used previous epidemiological knowledge in the choice of the candidate variables, and did not merely select predictors based on p-value from a list of irrelevant variables.

Our manuscript is in accordance with the ASA statement in several respects:

– “Scientific conclusions [...] should not be based only on whether a *p*-value passes a specific threshold.”
– “A *p*-value, or statistical significance, does not measure the size of an effect or the importance of a result.”
– “By itself, a *p*-value does not provide a good measure of evidence regarding a model or hypothesis.”

Besides providing the p-values for the statistical tests employed, we also showed the magnitude of the associations (the odds ratios with confidence intervals), and compared the model’s predictive performance in several ways (including ROC curve, discrimination slope, accuracy, Matthews correlation coefficient between the actual and predicted status, and reclassification tables). Unlike the example chosen by Nuzzo in her paper, “Statistical errors” (*Nature*, vol. 506, 2014) regarding some “actually tiny” effects reported elsewhere, the magnitude of association reflected by the odds ratio figures in our manuscript (medians between approximately 3.6 and 7) is not small. Neither is the net reclassification improvement of approximately 15%. Although each of these model performance measures features debatable merits if analyzed in isolation, they all suggest the same interpretation: that the expression of personal beliefs about the effects of smoking is associated with smoking behavior. Not only is this association intuitive, but it is also in line with the results of studies by Jester et al., Wellman et al., and Krosnick et al., cited in Section 4 of the manuscript.

## 3. Results

This study included 275 children and adolescents from the social protection system, 155 from the PMA system and 120 from the residential system, including family-type homes and day shelters. The responsiveness rate of the study was 75.24%.

The first part of Section 3 in our manuscript is mainly descriptive and focuses on the demographic descriptors and the answers to the questionnaire items. 

Next, we performed a multivariate analysis in order to find the variables associated with smoking in foster care teenagers. Observing that the expression of the study participants’ personal beliefs about the effects of smoking was associated with smoking, we wanted to verify whether that expression may offer additional predictive value relating to smoking, compared to classical demographic factors.

The age of the girls was higher than that of the boys (15 vs. 13 years, respectively, *p* = 0.018) and overall higher in participants from the residential system than in those from PMA (*p* = 0.001). The gender distribution was similar in the two social-care systems. Smoking habit was more frequent in the residential system than that in the PMA (*p* < 0.001), as were smoker tutors or parents (52.7% vs. 32.9%, *p* < 0.001) and exposure to secondhand smoking (46.4% vs. 31.8%, *p* < 0.001). The p value was calculated using the Wilcoxon rank-sum test for age, and chi^2^ for the categorical variables.

In total, 62 participants (22.5%) declared themselves as current smokers. The estimated number of daily consumed cigarettes was not associated with gender (*p* = 0.200), but tended to be higher in participants from the residential system than in those from the PMA: 7.5 (interquartile range: 3–15) vs. 3.5 (interquartile range 2–7.5), *p* = 0.058. It was not associated with the smoking status of the participant’s parents or tutors (*p* = 0.329). The estimated number of cigarettes consumed per day was positively associated with age (*rho* = 0.448, *p* < 0.001), but not with the declared age at which the participants began to smoke regularly (*p* = 0.805). Participants from the residential system were more likely to be daily smokers than those from the PMA (OR = 4.13, *p* = 0.026).

**Figure 1:**
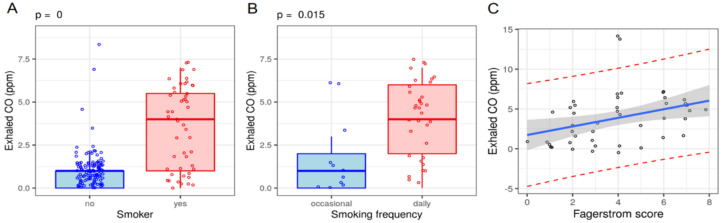
Dependency of exhaled CO on (**A**) smoking status, (**B**) smoking frequency, and (**C**) Fagerström score.

Smoker status was confirmed by dosing CO in the exhaled air, and the degree of dependence was assessed using the Fagerström score. The mean CO value in the smokers was 4 ppm (interquartile range: 1–6 ppm), significantly higher than in the nonsmokers (*p* < 0.001). Two participants who declared themselves nonsmokers and two former smokers demonstrated CO values greater than 2.5 ppm and higher than those in the rest of their respective subgroups. Exhaled CO was not influenced by general demographic factors (gender, *p* = 0.533; foster care system, *p* = 0.090), but was associated with frequency of smoking (median CO of 5 ppm in daily smokers vs. 1 ppm in occasional smokers, *p* = 0.015) and positively correlated with the estimated number of cigarettes consumed daily (*rho* = 0.348, *p* = 0.015), as well as with the Fagerström score (*rho* = 0.448, *p* = 0.002) (Figure 1). The average Fagerström score was 4 (interquartile range: 2–6). It was not influenced by gender or environment. Participants who declared that they smoked on a daily basis also demonstrated a higher Fagerström addiction score than occasional smokers (*p* = 0.009).

Among the current smokers, 35/61 (57.4%) declared that their parents or tutors were aware of their smoking habit, and 18/60 (30%) said that their parents agreed with them smoking. Of 60 respondents, 46 (76.7%) claimed to be exposed to secondhand smoke, most of them at school (58.1%) and in the family (35.5%). The most frequently invoked reason for smoking was peer group (“People around me smoke”, 38.7%), followed by the need for relaxation (“Smoking helps me relax”, 35.5%). Regarding the way in which the participants procured tobacco products, an alarmingly high proportion (36/57, i.e., 63.2%) declared they were able to buy them from the store, despite the fact that selling tobacco to minors is prohibited by law; 19.3% of them received their cigarettes from friends, and 12.3% asked friends to buy cigarettes for them, while only 5.26% stole cigarettes from parents/tutors. Most current smokers (36/57, 63.2%) had previously attempted at least once to quit smoking. Of 62 respondents, 58 (93.5%) admitted that they were aware of the health risks associated with smoking, and 54/60 (90%) knew that smoking was also harmful “to others”. Among the sources that inform teenagers regarding the effects of smoking, school was mentioned by most of the respondents (37/60, 59.7%), followed by the media (31/60, 50%) and family (25/60, 40.3%).

Given the known harmful effects of smoking, especially if started at a young age, we aimed to identify the predictors of smoker status in foster care teenagers. Identifying people at risk of starting smoking may enable more focused counseling and closer follow-ups of smoking cessation strategies in this particular population.

The preliminary selection of smoking status predictors, performed by univariate analysis and the exclusion of predictors with low probability (<50%) of association with the outcome, is shown in Table 1.

In the analysis, former smokers were included in the group of nonsmokers. All the participants were considered to be smokers or nonsmokers on the basis of their declared status on the questionnaires and not on their exhaled CO values. Following a multivariate analysis with backward selection, we observed that factors that maintain their predictive significance for the current smoking status in foster care teenagers were older age, male gender, residential type of foster care system, previous exposure to passive smoking, and the absence of the belief that smoking exerts harmful effects; the belief that quitting smoking created positive effects was negatively associated with smoking (Table 2).

Observing that the independent predictors for current smoking status included both general demographic characteristics and the personal opinions of the participants related to smoking, we assessed the practical relevance of the latter, reflected by their incremental predictive value compared to classical demographic factors. We thus compared two predictive models for current smoking status: a basic model (including age, gender, type of foster care system, and previous exposure to passive smoking) and the complete model that, in addition to the basic model, included the lack of belief that smoking was harmful and the belief that quitting smoking created beneficial effects, as expressed by the participants in the questionnaire.

**Figure 2:**
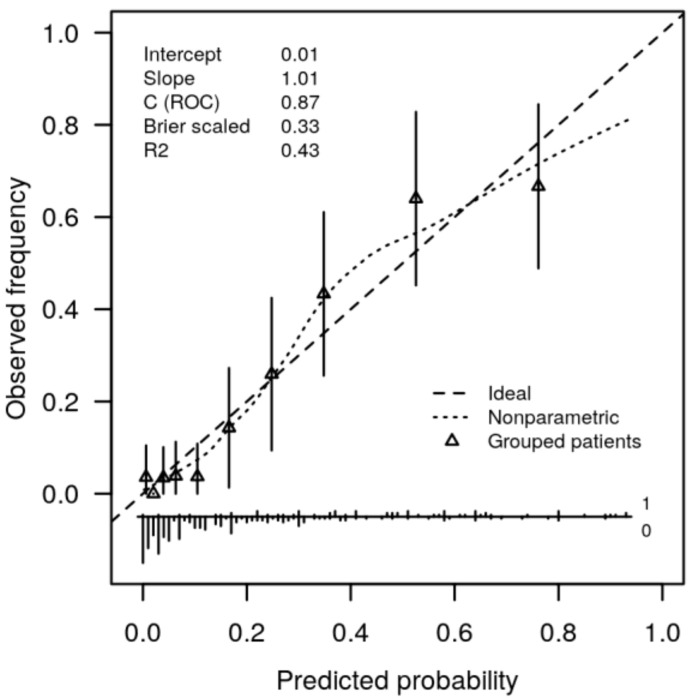
Validation plot for complete model. Correspondence between predicted probabilities and observed frequencies of current smoking in the studied participants, sub-grouped by deciles of predicted probability. Triangles and whiskers show average and 95% confidence intervals for observed frequencies of actual smoking in each subgroup (bottom). Spikes represent participant distribution according to individual predicted probability for smoking status (1 = smoker, 0 = nonsmoker).

The complete model demonstrated higher predictive value than that of the basic model (*p* < 0.001, likelihood ratio test). The complete model, which included the expressed opinion of participants regarding the effects of smoking, offered predictions on their current smoking status that appropriately matched the observed distribution of this feature in the studied group (*p* = 0.347, Hosmer–Lemeshow goodness-of-fit test) (Figure 2).

**Figure 3:**
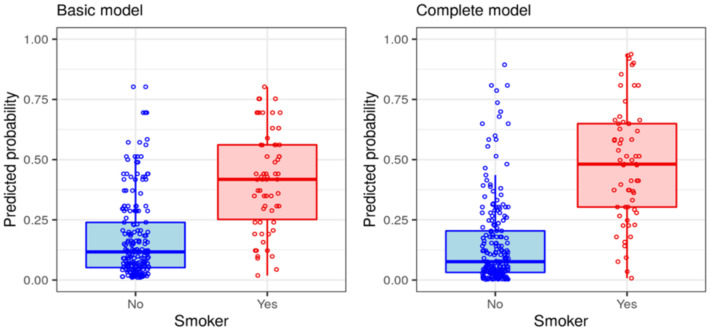
Predicted probabilities in smokers and nonsmokers, calculated by the (**left**) basic and (**right**) complete models. Boxplots show median probabilities (solid horizontal lines), interquartile ranges (boxes), and 95% confidence intervals for medians (whiskers). Individual values shown in open circles.

According to the complete model, the calculated probability of being a smoker was less than 20.4% in 75% of nonsmokers, whereas 75% of actual smokers displayed a predicted probability higher than 30.3% (Figure 3). 

**Figure 4:**
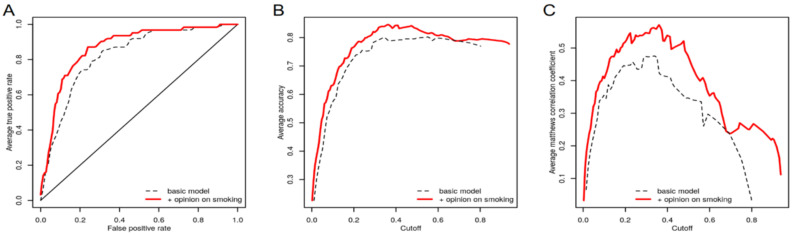
Comparison of performance measures of complete vs. basic predictive model for smoking behavior in foster care teenagers. (**A**) Receiver operating characteristic (ROC) curve. (**B**) Average accuracy depending on cut-off. (**C**) Average Matthews correlation coefficient depending on cut-off.

The discrimination slope, calculated as the difference between the average predicted probabilities in smokers and nonsmokers, respectively, increased from 0.24 in the basic model to 0.33 in the complete model. The complete model provided good discrimination between smoker and nonsmoker teenagers, with an area under the receiver operating characteristic curve (i.e., *c* statistic) of 0.870 (CI95: 0.821–0.919), higher than that in the basic model, where it was *c* = 0.824 (CI95: 0.769–0.880) (Figure 4A). Other performance measures, including accuracy and the Matthews correlation coefficient between the actual and predicted status, were better in the complete than those in the basic model for the entire range of the considered cut-off values (Figure 4B,C).

Although several computed parameters supported the higher predictive value of the complete model, a more intuitive way of comparing it with the basic model was to examine changes in the classifications that occurred when predicted probabilities of smoking behavior took into account the expressed point of view of the participants on the effects of smoking. For instance (Table 3), we chose a probability cut-off value of 36%, corresponding to the maximal value of the Matthews correlation coefficient for the complete model. Among smokers, the complete model reclassified 12 participants in the correct direction (they were considered to be low-risk by the basic model, with a calculated risk of smoking lower than the 36% threshold, but they were de facto smokers), and 5 participants in the incorrect direction (they were actual smokers, included in the high-risk group by the basic model and in the low-risk group by the complete model). The net reclassification in smokers was thus 7/62, resulting in an 11.3% increase in sensitivity. Similarly, the net reclassification in nonsmokers was 9/212, equaling an increase in specificity of 4.25%. The net reclassification improvement for the chosen threshold was 15.54% (CI95: 2.19–28.87%, *p* = 0.022).

Not limiting to a single threshold, the integrated discrimination improvement, calculated as the average of sensitivity and 1-specificity over the entire interval of possible threshold values (between 0 and 1), was 8.80% (CI95: 4.33–13.26%, *p* = 0.0001).

## 4. Discussion

As in the study conducted by Ahmadi-Montecalvo [17], teenagers from the PMA system were at a lower risk of becoming smokers than those from the residential system. The percentage of smokers in the residential system was approximately threefold higher than that in the PMA. There were further differences between the two foster care systems in terms of the age of smoking onset and the number of cigarettes consumed daily: those coming from the residential system initiated smoking earlier and used to smoke more cigarettes per day, thus reflecting less healthy behavior and weaker supervision compared to those in the professional maternal care system [7,18,19]. Similarly to Svetlana Schpiegel et al., who studied an important group of subjects from the institutional environment, we observed an association between male gender and smoking status [9]. In our study, we assessed the degree of nicotine dependence and risk factors for smoking in a large number of foster care teenagers from two different counties in Romania.

Compared with data from GYTS Romania (Global Youth Tobacco Survey, GYTS Romania), where 35.5% of students were exposed to secondhand smoke at home, in our study, the overall percentage of exposure to secondhand smoke was higher (46.4%), with significant differences depending on the environment of origin (higher rates in participants from the residential system than in those from PMA, 64.7% vs. 31.8%) [20]. A possible explanation for the higher reported secondhand smoke rate in our research was the absence of legal tutors during the completion of the questionnaires and, consequently, less pressure on participants to hide the reality and provide “correct” answers.

Age and exposure to secondhand smoke are additive risk factors [21,22,23,24]. For those who come from the social protection system, the risk of becoming smokers increases at an older age. School was the source of information that most respondents preferred [25]. The knowledge that smoking is harmful to health and the awareness of the effects of smoking are negative predictors that reduce the likelihood of young people becoming smokers [4,6,8,25].

A meta-analysis conducted on a large population of adolescents in 1984–2015 identified other categories of predictive factor for initiating smoking. Positive predictors were age, ethnicity, single-parent families, personality or temperamental disorders, depression, attention deficit, exposure to family smoking, susceptibility to marketing products, and poor social status, whereas negative predictive factors were high academic level, respiratory diseases, the inclusion of antismoking education in school curricula, a high level of self-esteem, and a close relationship with parents [26]. Given that minors in the social protection system often meet many of the conditions that favor the initiation of smoking, campaigns to identify and combat positive predictors must be supported in the long term [27].

The risk of becoming a daily smoker increases significantly in the first 3 years after starting smoking. It seems that for girls, family factors are decisive, while for boys, the school environment and peers are more important [6]. In our research, the adolescents came from the social protection system and were deprived of the comfort of the traditional family. Along with this major positive predictor for initiating and continuing smoking, a significant percentage were from ethnic minorities, and others had suffered various psychological traumas, which makes smoking cessation campaigns even more difficult.

Both the CO in the exhaled air and the Fagerström score are parameters that assess the degree of smoking addiction. The highly significant correlation between exhaled CO and the value of the Fagerström score confirmed the importance of these parameters in terms of the correct and real assessment of smoker status and degree of addiction [28].

The expressed opinion regarding the effects of smoking on health was clearly associated with smoking status in foster care teenagers. The absence of the belief that smoking created harmful effects and the belief that quitting smoking created positive effects showed significant incremental predictive value for smoking status over classical demographic factors. Although several psychological features, such as externalizing and internalizing behavior [11], rebelliousness, susceptibility to smoking, and the intention to smoke in the future [26] were associated with smoking in youth, these factors were not studied in the specific population of foster care teenagers. The expressed opinion of these individuals, without the inhibitory presence of legal tutors, seems to reflect their choice to smoke. The under-representation of the health risks of cigarette smoking also concerns the general population [26,29].

In terms of the external validity of our findings, the signs of the associations between individual predictors and smoking were similar to those found in other, independent studies (as presented above). For example, age, male gender, residential foster care system, previous exposure to passive smoking, and the lack of belief that smoking is harmful were all positively associated with smoking in the studied group of foster care teenagers. Meanwhile, the belief that quitting smoking creates beneficial effects is negatively associated with smoking. The large values of the calculated odds ratios are indicators of the strength of these associations.

The limitations of this study include its cross-sectional design and the self-reporting nature of the questionnaire, with the respondents able to report data with less accuracy. The participants may also have under-reported their smoking status due to their age. Another limitation of this study is that the sample was restricted to only two counties from Romania and may not be representative of all foster care adolescents in our country. We did not explore other factors, such as mental health or delinquency, because we performed a study on the general population, and we included all foster care adolescents who expressed their consent. A further investigation of predictors for the onset of smoking in foster care teenagers in a longitudinal study should provide a deeper understanding of this complex phenomenon.

This study is relevant because institutionalized teenagers initiate tobacco use and other delinquent behavior at a younger age than the general population [10]. Thus, prevention programs should be initiated as early as possible and adapted to their particular social conditions. The need to prevent or delay tobacco use must be supported, as smoking can be a launching pad for the discovery and use of other drugs [30,31].

## 5. Conclusions

In addition to age, gender, social environment, previous exposure to secondhand smoking, and residential type of foster care system, the expressed opinions regarding the health effects of tobacco use are associated with smoking in foster care teenagers. The prediction of smoking behavior may enable more effective, better-adapted and more efficient smoking-prevention programs in vulnerable populations. This study revealed statistically significant linear correlations between CO value and Fagerström score. The correlation was representative of the entire study population. The correlation of CO values in the exhaled air was statistically highly significant with smoking status, the frequency of smoking, and the number of cigarettes consumed daily. Age and environment of origin exerted a cumulative effect as positive predictors of smoking.

## Figures and Tables

**Table 1:** Univariate analysis of potential predictors ^a^ for current smoker status in foster care teenagers.

Predictor	All Participants	Nonsmokers	Smokers	*p* Value
	*n* = 274 ^b^	*n* = 212	*n* = 62	
Age ^c^	14.0 (12.0; 16.0)	13.0 (12.0;15.0)	15.0 (14.0;17.0)	<0.001
Foster care system:				<0.001
PMA	154 (56.2%)	136 (64.2%)	18 (29.0%)	
residential	120 (43.8%)	76 (35.8%)	44 (71.0%)	
Gender:				0.005
m	133 (48.9%)	93 (44.1%)	40 (65.6%)	
f	139 (51.1%)	118 (55.9%)	21 (34.4%)	
Smoker parents or tutors:				<0.001
Yes	145 (52.9%)	96 (45.3%)	49 (79.0%)	
No	129 (47.1%)	116 (54.7%)	13 (21.0%)	
“I believe that quitting smoking has the following effect”:				0.219
Positive	126 (49.0%)	102 (52.0%)	24 (39.3%)	
Do not know	104 (40.5%)	75 (38.3%)	29 (47.5%)	
Negative	27 (10.5%)	19 (9.69%)	8 (13.1%)	
“I believe that smoking is harmful”:				0.003
Yes	242 (91.0%)	192 (94.1%)	50 (80.6%)	
No	24 (9.02%)	12 (5.88%)	12 (19.4%)	
“I am aware of the risks related to smoking”:				0.2
Yes	263 (96.0%)	205 (96.7%)	58 (93.5%)	
No	9 (3.28%)	5 (2.36%)	4 (6.45%)	
Exposure to passive smoking:				<0.001
Yes	123 (46.2%)	77 (37.4%)	46 (76.7%)	
No	143 (53.8%)	129 (62.6%)	14 (23.3%)	
“Smoking is harmful to others”:				0.237
Yes	242 (93.4%)	188 (94.5%)	54 (90.0%)	
No	17 (6.56%)	11 (5.53%)	6 (10.0%)	
“I believe that electronic cigarettes are harmful”:				0.025
Yes	160 (80.4%)	121 (82.9%)	39 (73.6%)	
No	11 (5.53%)	4 (2.74%)	7 (13.2%)	
Do not know	28 (14.1%)	21 (14.4%)	7 (13.2%)	

Note: ^a^ Figures shown for all predictors, with the exception of age, are: number of answers (percentage) for each category. ^b^ Out of 275 participants, only 274 were included in the analysis (one response regarding the smoker status was missing). ^c^ Age description is shown in years, as average (confidence interval 95%).

**Table 2:** Predictive factors for current smoker status in foster care teenagers (results of multivariate analysis).

Factor	Odds Ratio (CI 95%)	*p* Value
Age	3.98 (1.94–8.17)	0.0002
Gender (male: female)	4.54 (2.13–9.69)	<0.0001
Foster care system (residential type)	3.62 (1.73–7.58)	0.0007
Previous exposure to passive smoking (reported by the participants)	5.00 (2.18–11.50)	0.0001
Lack of belief that smoking is harmful (answer in the questionnaire)	6.96 (2.23–21.69)	0.0008
Belief that quitting smoking has beneficial effects (answer in the questionnaire)	0.33 (0.15–0.73)	0.0059

**Table 3:** Reclassification table of study participants stratified for events (smokers) and nonevents (nonsmokers).

Current Status	Basic Model	Complete Model	
		≤36%	>36%
Nonsmoker (*n* = 212)	≤36%	175	5 ^a^
	>36%	14 ^b^	18
Smoker (*n* = 62)	≤36%	14	12 ^b^
	>36%	5 ^a^	31

^a^ = reclassification in the incorrect direction, ^b^ = reclassification in the correct direction.
